# The Emergence of Self‐Awareness in Preterm Infants: Insights From the Rooting Reflex

**DOI:** 10.1111/apa.70453

**Published:** 2026-01-20

**Authors:** Alessia Touraton, Fleur Lejeune, Frédérique Berne Audéoud, Thierry Debillon, Marie Chevallier, Edouard Gentaz, Julia Doutau

**Affiliations:** ^1^ Intensive and Regular Neonatal Care Unit CHRU Grenoble Alpes Grenoble France; ^2^ Sensorimotor, Affective and Social Development Unit, FPSE University of Geneva Geneva Switzerland; ^3^ CNRS Grenoble France

## Abstract

**Aim:**

Touch is the first sense to develop during fetal life. Sensory exploration in preterm infants may underlie early sensory self‐awareness. This study aimed to demonstrate the emergence of sensory self‐awareness in preterm infants by analysing behavioural responses of the rooting reflex.

**Methods:**

This single‐centre, prospective study included preterm infants born between 24 and 37 weeks gestational age (wGA). The rooting reflex was assessed via video in two conditions: external stimulation and facilitated self‐stimulation. The rooting reflex was characterised by four progressively complex behavioural responses. The primary outcome was the relative frequency of these behaviours, compared between the two stimulation types.

**Results:**

Twenty‐seven children were included (mean gestational age: 30.5 wGA; mean birth weight: 1416 g). Behaviour 3 (mouth opening and/or tongue protrusion without head movement) was significantly more frequent during self‐stimulation than external stimulation (35.7% vs. 24.9%, *p* = 0.005). No significant differences were observed for other behaviours.

**Conclusion:**

The rooting reflex response in preterm infants depends on stimulation type, with a preference for self‐stimulation. This suggests an early ability to distinguish self from non‐self, supporting the idea that the rooting reflex reflects implicit sensory self‐awareness. These findings offer novel insights into preterm infant sensory processing and developmental care.

**Trial Registration:**

Favourable opinion from the committee for the protection of individuals CPP N2023‐A02220‐45. Favourable opinion of Clinical Trial: NCT NI38RC23.0354

## Summary

- Early sensory self‐awareness in preterm infants is crucial for understanding neonatal development and improving care.
- Infants born between 24 and 37 weeks of gestational age demonstrate tactile discrimination and a preference for self‐stimulation.
- These results suggest that sensory self‐awareness provides a foundation for cognitive and social development, offering important insights for clinical practice and informing future research on neonatal sensory and neurological maturation.

**Abbreviations**
NICU: neonatal intensive care unit
wGA: weeks of gestational age

## 1 Introduction

Touch may contribute to the emergence of self‐awareness, initially conceptualised as a perceptual experience of the body. From the first weeks of life, newborns develop an implicit understanding of their bodies as active, situated, and distinct entities—referred to as an *ecological self* [1, 2, 3]. This foundational self‐awareness is supported by sensoriality and evolves through interactions with the physical and social environment. It seems to follow maturation of the different senses. Newborns can distinguish between their own cries and those of other infants, and neonatal crying can elicit distress in other newborns [4, 5, 6]. By 9 months, infants begin to demonstrate joint attention and social expectations [7]. Around 14 months, mirror self‐recognition emerges, and by 18 months, infants begin to engage in collaborative actions and develop a rudimentary co‐consciousness, integrating the perspective of others [8]. By age two, this awareness is evidenced by behaviours such as embarrassment in front of a mirror, indicating an explicit self‐consciousness shaped by social interaction [9].

As touch is the first sense to develop during fetal life, tactile sensoriality may serve as an initial step toward self‐awareness [10, 11]. Even preterm infants exhibit early tactile competencies [12], expressed through neonatal reflexes, which are not merely passive, stereotyped responses but convey real sensory information. It is shown that the grasping reflex may reflect early tactile processing abilities, including discrimination and memory, even in very preterm neonates [12, 13, 14]. Other neonatal reflexes—such as the rooting reflex, which is characterised by head turning and mouth opening in response to perioral stimulation—provide an interface for sensory exploration [2]. In a seminal experiment by Rochat and colleagues, [2] full‐term newborns were observed during spontaneous self‐touch (e.g., a hand contacting their own cheek) and in response to external touch delivered by an experimenter. Microanalyses of the newborn's behaviour revealed a significant difference in rooting responses showing more pronounced rooting to external touch than to self‐generated touch, indicating an early capacity to distinguish between self‐ and non‐self stimulation. These findings support the idea that a rudimentary form of self‐awareness emerges from birth through tactile sensoriality. To date, no such studies have been conducted in preterm infants. However, few studies assessing feeding in premature neonates suggest the presence of the rooting reflex from 29 wGA [15].

In Neonatal Intensive Care Unit (NICU), where sensory exposure is often intense and poorly adapted, recognising the importance of touch in shaping early self‐awareness is essential for guiding developmental care [16]. Despite early evidence of sensory competence in preterm neonates, the development of self‐awareness processing remains underexplored.

The primary objective of this study is to assess the emergence of sensory self‐awareness in preterm infants by exploring their tactile responses via the rooting reflex. We hypothesize that preterm infants will exhibit a stronger response to external tactile stimulation compared with facilitated self‐stimulation, indicating an early capacity to differentiate between self and non‐self.

## 2 Method

### 2.1 Participants

This single‐center, prospective, observational, and comparative study was conducted in a level III NICU at Grenoble Alpes University Hospital between April 2024 and March 2025. Preterm neonates born between 24 and 37 weeks of gestational age (wGA) and aged at least 3 days at the time of inclusion were eligible for enrollment. Neonates were excluded if they required respiratory support except nasal canulae. Those with congenital malformative syndromes or known genetic disorders, presenting with neurological complications, such as grade III or IV intraventricular haemorrhage according to Papille classification [17] or periventricular leukomalacia, receiving sedative treatments, including opioids or clonidine, aminergic treatment, or presenting with an acute condition—such as shock, sepsis, acute cardiac or respiratory failure—were also excluded.

### 2.2 Procedure

The procedure consisted of brief perioral tactile stimulation, performed under specific medical conditions and only when the newborn was in a state of spontaneous calm wakefulness (stage 4 on the Brazelton scale) [18]. It was never initiated if the infant was asleep or agitated and was interrupted if the infant fell asleep or began to cry. The stimulation was performed in the incubator or cradle, before or apart from feeding, and always in accordance with the infant's natural care rhythm to avoid any disruption. Infants were never woken specifically for the procedure. Each session lasted approximately 5–10 min and was conducted by one paediatrician from the neonatal department. Parents could be present in the room but were asked to remain outside the infant's field of vision and to stay silent. The entire procedure was video recorded.

The pediatrician positioned the infant in the supine position, using the standard motor support cocoon typically employed for preterm neonates. This positioning allowed free movement of the upper limbs toward the face. The head was maintained in a midline position, free to turn to either side, and lightly supported by the experimenter. Clothing did not restrict limb movement, and infants were not swaddled. Each infant underwent external stimulation and facilitated self‐stimulation. These two stimulation sessions were separated by a minimum of 2 h and a maximum of 48 h. The order of stimulation type was randomly assigned.

Each stimulation session began and ended with a 30‐s baseline observation period. The stimulation then consisted of approximately five trials, each lasting between 8 and 16 s, and was followed by a 30‐s rest. The side of stimulation (right or left) was determined randomly, and sides alternated thereafter. In the external stimulation condition, the experimenter gently touched the corner of the infant's mouth with the tip of their index finger, performing five light touches during each trial. In the facilitated self‐stimulation condition, the experimenter gently guided the infant's hand toward their mouth to initiate contact at the corner of the lips, then slid the hand outward along the cheek, and this gesture was repeated five times. This gesture was supported as needed to compensate for hypotonia.

### 2.3 Measures

#### 2.3.1 Medical Data

The perinatal data were collected prospectively from the child's medical record by the paediatrician and included gestational age at birth, birthweight, small for gestational age below 10th percentile [19], intrauterine growth restriction (corresponding to a break in growth [20]), antenatal corticosteroid therapy, mode of delivery, APGAR scores at 5 and 10 min, spontaneous or induced prematurity, and type of feeding. The age at achievement of full oral feeding autonomy (defined as complete cessation of enteral tube feeding), comorbidities like bronchopulmonary dysplasia defined as respiratory support for more than 28 days (according to Bancalari and Jobe) [21], all type of retinopathy of prematurity to cover all forms of damage from stage 1 onwards (according ICROP) [22] and ulceronecrosing enterocolitis (all stages according to Bell classification) [23] were also recorded. Data collected at the time of each stimulation session included postnatal age, weight on the day of the test, the number of other procedures performed on the same day (such as an ultrasound scan, an eye examination, an incubator change, a bath, a blood test, or an electroencephalogram), the presence of nasal cannula, the presence of nasogastric tube and its location (mouth or nose), a qualitative analysis of sucking with the stage assessed according to the Bross score, and the number of skin‐to‐skin sessions during the 7 days preceding the stimulation.

#### 2.3.2 Behavioural Measures

Behavioural coding was performed based on video recordings. Four cumulative behaviours were observed, meaning that if behaviour 4 was present, the preceding three behaviours were also observed. Although behaviour 3 was already described in the existing classification [2], we added a supplementary category within this behaviour 3, based on a classification used by Nyqvist et al. reflecting a developmentally adapted expression of the reflex in infants with limited head control, such as preterm infants [15]. The behaviours were defined as follows [2]:

– Behaviour 1: Head movement outward or toward facial stimulation
– Behaviour 2: Head movement toward the stimulation
– Behaviour 3: Head movement away from or toward the facial stimulation with an open mouth, a slightly open mouth, or tongue protrusion, or mouth opening, similar to a sucking motion [15]
– Behaviour 4: Head movement toward facial stimulation with an open mouth, a slightly open mouth, or tongue protrusion

For each infant and each condition, the presence or absence of each of the four behaviours was recorded every second. The relative frequency of each behaviour was calculated as the number of seconds during which the behaviour was observed, divided by the total duration of the episode. The frequencies were then averaged across episodes for each behaviour, each infant, and each condition. Finally, relative frequencies were expressed as percentages.

Inter‐rater reliability analysis was conducted on eight videos (15% of the total dataset) between a master coder and a naïve coder from the study, following joint training on four preliminary coding videos. An Intraclass Correlation Coefficient analysis was performed to assess reliability, yielding a Cronbach's alpha of 0.99, indicating excellent agreement between raters.

#### 2.3.3 Primary Outcome

Primary outcome was the occurrences for each behaviour relative to rooting reflex compared between external stimulation and facilitated self‐stimulation, expressed as percentages calculated as the number of seconds during which the behaviour was observed divided by the total duration of the episode.

### 2.4 Sample Size Calculation

An a priori power analysis was conducted using G*Power 3.1.9.7 software. Based on a matched‐pair design, with an expected effect size of 0.80, a power of 0.5, and a significance level (*α*) of 0.05, the required sample size was calculated to be 19 participants. Given that the statistical significance of the results obtained with these 19 participants was close to the threshold (*p* = 0.05), we decided to include an additional 10 infants. This extension aimed to strengthen the robustness of the findings and ensure more reliable and conclusive results.

### 2.5 Statistical Analysis

Analyses were conducted using IBM SPSS Statistics (version 29). For continuous variables, paired Student's *t*‐tests were applied when the distribution of within‐subject differences was approximately normal, while Wilcoxon signed‐rank tests were used when non‐normality was evident. For categorical variables, McNemar's test was used for paired comparisons. Pearson correlations were conducted to explore potential associations between the preference for one condition over the other and various variables, including gestational age, postnatal age, C‐section birth, and the Bross score. To calculate the preference score, the relative frequency difference between self‐stimulation and external stimulation was determined. Statistical significance was set at *p* < 0.05.

### 2.6 Ethics Statement

This study is a type 3 trial under the Jardé law, involving human participants. It was approved by the Committee for the Protection of Human Rights and Fundamental Freedoms (PREMATACT, reference 2023‐A02220‐45), the ethics committee of Grenoble University Hospital, and the French National Data Protection Authority. The clinical trial was registered under the identifier NCT ID 38RC23.0354. The study was conducted in accordance with the Declaration of Helsinki. Written informed consent was obtained from the parents of all participating infants.

## 3 Results

**FIGURE 1:**
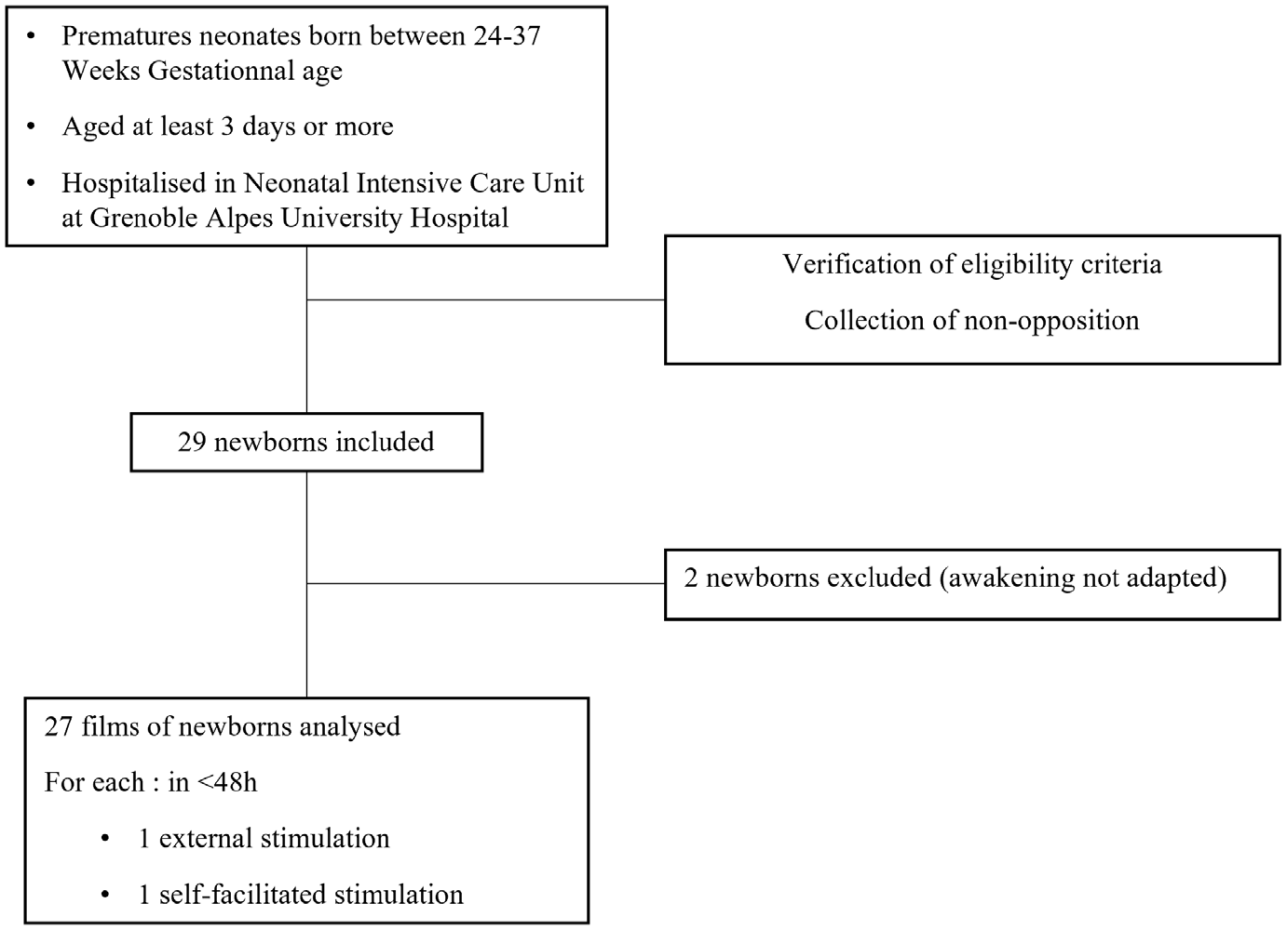
Flow chart.

Twenty‐nine preterm infants were initially enrolled in the study. The test results for two participants were excluded due to sleep‐related issues, rendering the data uninterpretable. The final sample comprised 27 preterm infants (Figure 1). Their perinatal data are presented in Table 1.

**TABLE 1:** Preterm infants' perinatal data.

Characteristics (*N* = 27)	*N*/M	%/SD [min; max]
Gender		
Girl	10	37%
Boy	17	63%
Gestational age (weeks)	30.6	3.1 [24.1; 34.7]
Birthweight (g)	1416	446 [623; 2365]
Small for gestational age	2	7%
Intrauterine growth restriction	4	15%
Antenatal corticosteroid therapy	25	93%
Caesarean delivery	19	70%
Apgar score < 7 at 5 min	2	7%
Apgar score < 7 at 10 min	0	0%
Spontaneous prematurity	9	33%
Feeding		
Breastfeeding	17	63%
Mixed feeding	2	7%
Formula feeding	8	30%
Gestational age at full oral feeding (weeks)	36,2	1.1 [34.3; 39.4]
Bronchopulmonary dysplasia^a^	14	52%
Retinopathy of prematurity^b^	6	22%
Necrotizing Enterocolitis	0	0%

Abbreviation: M, mean.

^a^ Bronchopulmonary dysplasia (according to Bancalari and Jobe) defined as respiratory support for more than 28 days.

^b^ We have used the term retinopathy of prematurity to cover all forms of damage from stage 1 onwards (according to ICROP).

The cohort included 17 boys (63%), antenatal corticosteroid therapy has been administered to 25 mothers (93%), and 19 infants (70%) were delivered by C‐section. Infants were born at a mean gestational age of 30 weeks and 4 days (SD = 3.1 weeks, range 24.1–34.7 weeks), with a mean birthweight of 1416 g (SD = 446 g, range 623–2365 g).

Regarding feeding, 17 infants (63%) received breastfeeding. The mean gestational age at full oral feeding was 36 weeks and 2 days (SD = 1.1 weeks, range 34.3–39.4 weeks). In terms of neonatal complications, 14 infants (52%) developed bronchopulmonary dysplasia (defined as respiratory support for more than 28 days) [21].

### 3.1 Medical Data Comparison

The medical data collected at the time of each stimulation session are presented in Table 2. Between self and external stimulation, neonates included were respectively 29.6 and 29.3 days old (*p =* 0.465), weighted 2004 and 1998 g (*p =* 0.754), 79% and 82% (*p =* 0.564) had nasogastric tube. No significant effect was found between the conditions.

**TABLE 2:** Preterm infants' medical data collected at the time of each stimulation session (self‐stimulation vs. external stimulation).

Characteristics	Self‐stimulation	External stimulation	*p*
	*N* (%) or M (SD)	*N* (%) or M (SD)	
Post‐natal age (day)	29.6 (20.9)	29.3 (20.3)	0.465
Weight at test (g)	2004 (411)	1998 (419)	0.754
Nasal cannula	15 (56%)	14 (52%)	0.317
Nasogastric tube	21 (79%)	22 (82%)	0.564
Other procedures on the same day	5 (19%)	7 (26%)	0.317
Bross score	1.85 (1.38)	1.85 (1.35)	1
Number of skin‐to‐skin sessions^a^	5.78 (1.89)	5.53 (2.15)	0.403

^a^ During the seven days preceding the stimulation.

### 3.2 Behavioural Data Comparison

**FIGURE 2:**
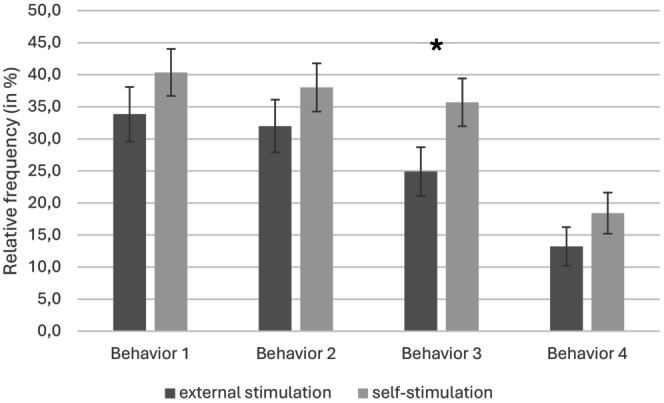
Relative frequency of each behaviour according to the two stimulation conditions. Behaviour 1: t (26) = 1.66, *p* = 0.054, *d* = 0.20; Behaviour 2: t (26) = 1.49, *p* = 0.074, *d* = 0.21; Behaviour 3: t (26) = 2.82, *p* = 0.005, *d* = 0.20; Behaviour 4: T = 211, *p* = 0.192, *r* = 0.10. *p < 0.5.

Figure 2 presents the relative frequencies of each behaviour across conditions. Behaviours 1 and 2 showed trends toward significance, with preterm infants tending to exhibit these behaviours more frequently during self‐stimulation (*M* = 40.4%, SD = 19.1; *M* = 38.0%, SD = 19.5) than during external stimulation (*M* = 33.8%, SD = 22.1; *M* = 32.0%, SD = 21.4). Behaviour 3 differed significantly between conditions, occurring more often during self‐stimulation (*M* = 35.7%, SD = 19.4) compared with external stimulation (*M* = 24.9%, SD = 19.7). No significant difference was observed for behaviour 4 (M = 18.4%, SD = 16.7; M = 13.2%, SD = 15.6) for self‐ and external stimulation, respectively.

### 3.3 Associations Between the Sense of Preference and Potential Explanatory Variables

Eighteen out of 27 infants had a positive preference score, indicating a preference for self‐stimulation, measured by behaviour 3. The results revealed no significant correlation between the preference score for behaviour 3 and the different variables: gestational age (*r* = 0.001, *p* = 0.995), postnatal age (*r* = −0.117, *p* = 0.562), and Bross score (*r* = −0.039, *p* = 0.847).

In addition, comparison of preference scores between infants born by caesarean section and those born vaginally showed no significant difference (*p* = 0.735).

## 4 Discussion

Infants experiencing a facilitated self‐stimulation—when their own hand was guided toward the perioral area—were more likely to show a differentiated orofacial response compared with when the same area was stimulated externally by an examiner, especially for the behaviour 3. These findings suggest the emergence of early sensory self‐awareness in preterm infants, as evidenced by differences in the expression of the rooting reflex depending on the source of tactile stimulation.

Following Rochat et al. we considered the reflex as a progression of integrated behaviours, with its full expression involving head turning, mouth opening, and tongue protrusion [2]. However, due to the axial hypotonia in preterm infants, an intermediate response (mouth opening and/or tongue protrusion without head movement, behaviour 3 adapted from Nyquist et al. [15]) was commonly observed [24]. This orofacial response was significantly more frequent during self‐facilitated stimulation, while the full reflex expression remained rare and showed no difference between conditions, likely due to neuromuscular immaturity (18.4% in the present study vs. 60% reported by Rochat et al. for the behaviour 4 in full‐term neonates) [2].

Contrary to our initial hypothesis that preterm infants will exhibit a stronger response to external tactile stimulation compared with facilitated self‐stimulation based on research by Rochat et al. our findings diverge: they studied full‐term neonates within their first 18 h of life and observed stronger rooting responses to external stimulation [2]. In contrast, our cohort of preterm infants (mean postnatal age of 29 days) had already experienced multiple perioral stimulations in NICU, which may explain the enhanced response to self‐facilitated touch. This pattern resembles Rochat's observations in 4‐week‐old full‐term infants, suggesting a developmental shift possibly related to emerging functional goals and learning processes [2]. Importantly, no significant correlation was found between postnatal age and rooting responses, suggesting that even limited ex‐utero experience may be sufficient to support early differentiation between self‐ and externally generated touch.

The differential expression of the rooting reflex suggests that early tactile reflexes may be shaped by sensory experience. In our NICU, newborns' hands are free and are positioned (skin‐to‐skin, postural and motor support with a cocoon) to allow hand‐to‐face contact, without exogenous perioral stimulation. To reduce confounding, assessments were conducted away from feeding times. Oral‐motor competence, evaluated using the Bross scale [25] showed no significant correlation with rooting preference, indicating that tactile responses were independent of feeding ability. These findings raise important questions about how early tactile discrimination contributes to the development of oromotor skills. Feeding, often delayed in preterm infants, can be accelerated through targeted oral stimulation from 26 to 29 wGA, emphasising that oral skills result from both neurological maturation and sensory learning [26, 27, 28, 29, 30]. Prior research emphasises the importance of adapting tactile stimuli to the infant's developmental stage [12], and our findings suggest that self‐directed touch may offer unique developmental benefits. Understanding how reflexes like rooting respond to sensory input could help optimise interventions, as self‐directed touch seems more physiological than external stimulations in supporting rooting reflex.

Our study adds to evidence that foundations of self‐awareness may emerge earlier than previously assumed, possibly even before birth, following the sequential maturation of the senses and initially supported by tactile sensoriality. In the third trimester, foetuses display organised behaviours such as preferential hand‐to‐mouth movements, enabling experiences of “double touch,” where the body is both source and recipient of tactile input [31, 32]. Our findings suggest that the rooting reflex may reflect not only feeding readiness but also an early form of implicit bodily self‐awareness, the ability to differentiate self‐generated from external stimulations. This foundational self‐awareness may underpin later explicit, conceptual self‐awareness [33]. Recognising these signs in preterm infants has clinical implications, reinforcing their individuality and the need for tailored developmental care. The social environment, including caregiver interaction, skin‐to‐skin contact, and responsive handling, also plays a crucial role, as early self‐awareness emerges from the interplay of biological, neurological, and social factors [33]. Future studies should explore how these dimensions interact in shaping the developing sense of self in both term and preterm infants.

This study's strengths include detailed behavioural coding and excellent inter‐rater reliability. Limitations include its monocentric design, small sample, and lack of blinding, which may limit the generalizability and warrant replication in larger, multicentric cohorts. Despite these limitations, the study provides novel evidence that even very young preterm infants may differentiate between self‐ and externally generated touch, potentially reflecting the earliest signs of sensory self‐awareness. These findings highlight the importance of considering the infant's active sensory experience in developmental care and early neurodevelopment.

## 5 Conclusion

The behavioural response of the rooting reflex is modulated by the type of stimulation in preterm infants, with a preferential response to self‐stimulation, known as ‘double touch’. This supports an early ability to distinguish self from non‐self and suggests the emergence of sensory self‐awareness. Such foundational self‐awareness may underpin the later development of explicit, conceptual self‐awareness, which enables core human capacities such as reflective thinking, learning, creativity, and social interaction. This innovative perspective on the sensoriality of the preterm newborns could significantly advance research in developmental care.

## Author Contributions

Alessia Touraton, conceptualized and designed the study, collected data, drafted the initial manuscript, and critically reviewed and revised the manuscript. Fleur Lejeune conceptualised and designed the study, carried out all the analyses, drafted the initial manuscript, and critically reviewed and revised the manuscript. Julia Doutau conceptualised and designed the study, coordinated and supervised data collection, drafted the initial manuscript, and critically reviewed and revised the manuscript. Edouard Gentaz and Frederique Berne‐Audeoud conceptualised the study, critically reviewed, and revised the manuscript for important intellectual content. Marie Chevallier and Thierry Debillon critically reviewed and revised the manuscript for important intellectual content. All authors approved the final manuscript as submitted and agree to be accountable for all aspects of the work.

## Funding

The authors have nothing to report.

## Conflicts of Interest

The authors declare no conflicts of interest.

## Data Availability

Deidentified individual participant data (including data dictionaries and the results) will be made available, in addition to study protocols, the statistical analysis plan, analytic code and the informed consent. The data will be made available upon publication to researchers who provide a methodologically sound proposal for use in achieving the goals of the approved proposal. The data will be available beginning 3 months and ending 5 years after following article publication. Proposals should be submitted to accueilrecherche@chu‐grenoble.fr. To gain access, data requestors will need to sign a data access agreement.
